# Metaproteomics and metabolomics analyses of chronically petroleum‐polluted sites reveal the importance of general anaerobic processes uncoupled with degradation

**DOI:** 10.1002/pmic.201400614

**Published:** 2015-08-27

**Authors:** Rafael Bargiela, Florian‐Alexander Herbst, Mónica Martínez‐Martínez, Jana Seifert, David Rojo, Simone Cappello, María Genovese, Francesca Crisafi, Renata Denaro, Tatyana N. Chernikova, Coral Barbas, Martin von Bergen, Michail M. Yakimov, Manuel Ferrer, Peter N. Golyshin

**Affiliations:** ^1^ Consejo Superior de Investigaciones Científicas (CSIC)Institute of CatalysisMadridSpain; ^2^ Department of ProteomicsUFZ – Helmholtz Centre for Environmental ResearchLeipzigGermany; ^3^ Center for Microbial CommunitiesDepartment of Chemistry and BioscienceAalborg UniversityAalborgDenmark; ^4^ Institute of Animal ScienceUniversität HohenheimStuttgartGermany; ^5^ Centro de Metabolómica y Bioanálisis (CEMBIO)Facultad de FarmaciaUniversidad CEU San PabloMadridSpain; ^6^ Institute for Coastal Marine EnvironmentCNRMessinaItaly; ^7^ School of Biological SciencesBangor UniversityGwyneddUK; ^8^ Department of MetabolomicsUFZ – Helmholtz Centre for Environmental ResearchLeipzigGermany

**Keywords:** Anaerobic, Crude oil, Hydrocarbonoclastic, Mediterranean Sea, Metabolomics, Microbiology

## Abstract

Crude oil is one of the most important natural assets for humankind, yet it is a major environmental pollutant, notably in marine environments. One of the largest crude oil polluted areas in the word is the semi‐enclosed Mediterranean Sea, in which the metabolic potential of indigenous microbial populations towards the large‐scale chronic pollution is yet to be defined, particularly in anaerobic and micro‐aerophilic sites. Here, we provide an insight into the microbial metabolism in sediments from three chronically polluted marine sites along the coastline of Italy: the Priolo oil terminal/refinery site (near Siracuse, Sicily), harbour of Messina (Sicily) and shipwreck of MT Haven (near Genoa). Using shotgun metaproteomics and community metabolomics approaches, the presence of 651 microbial proteins and 4776 metabolite mass features have been detected in these three environments, revealing a high metabolic heterogeneity between the investigated sites. The proteomes displayed the prevalence of anaerobic metabolisms that were not directly related with petroleum biodegradation, indicating that in the absence of oxygen, biodegradation is significantly suppressed. This suppression was also suggested by examining the metabolome patterns. The proteome analysis further highlighted the metabolic coupling between methylotrophs and sulphate reducers in oxygen‐depleted petroleum‐polluted sediments.

**Abbreviations**
EPA: Environmental Protection Agency
FID: flame ionisation detector
HAV: Haven tanker
LTQ: linear trap quadrupole
MES: Messina
MPS: metaproteome source
PAH: polyaromatic hydrocarbon
Pet Hyd: total petroleum hydrocarbon
PRI: Priolo
TERCH: total extracted and resolved hydrocarbons
UPLC: ultra performance liquid chromatography

## 1 Introduction

Anthropogenic crude oil discharge into seawater, together with the chemical diversity of crude oil components and environmental constraints such as depth, temperature, O_2_ concentration and nutrient input, have been commonly reported to distinctly influence the large‐scale distribution of microbial populations and the geochemical and biodegradation processes the populations mediate 1, 2, 3, 4. The relative abundance of bacteria specialised in the degradation of alkanes and polycyclic aromatic hydrocarbons (PAH) and the global gene expression are modulated by variations in the crude oil inputs in the sea 5, 6, 7, 8, 9. These effects have been reported for bacteria of the genera *Alcanivorax*, *Marinobacter*, *Oleispira*, *Thalassolituus*, *Oleiphilus*, *Cycloclasticus* and *Neptunomonas* 5, 9, 10 and for selected catabolic genes, e.g., those encoding alkane monooxygenases, catechol 1,2‐dioxygenases, catechol 2,3‐dioxygenases, naphthalene dioxygenase components, and genes relevant to carbon, nitrogen, phosphorous, sulphur and iron cycling 1, 2, 3, 12. Recent studies of the Deepwater Horizon oil spill have further revealed that such shifts occurred within 1 month of the spill 13.

Compared to sites in which accidental crude oil spills occurred, such as the Gulf of Mexico 13 or the *Prestige* Tanker accident off the NW coast of Spain 14, the Mediterranean Sea has been generally neglected by the international research community regarding studies on marine oil pollution. However, this area hosts large numbers of pipeline terminals, oil refineries and offshore platforms and 20% of global crude oil traffic 15, 16 with numerous reported tanker accidents 17, 18. The scale of the pollution in the Mediterranean is severe, despite Mediterranean Sea only represents approximately 1% of the total sea surface of the planet. Several studies and reports have also demonstrated that selected areas in the Mediterranean Sea are polluted with toxic compounds other than crude oil components 19, leading to a synergistic increase in the overall toxicity 20. Additionally, compared to open oceanic areas, the Mediterranean Sea is a semi‐enclosed basin, and the chemical species are trapped and tend to accumulate rapidly because the theoretical flushing time of Mediterranean water takes as long as approximately 70–90 years.

Although enhanced crude oil inputs in the Mediterranean Sea basin may sustain versatile microbial populations, the distribution and metabolic potential of these populations (in the context of physico–chemical conditions such as the water temperature, O_2_ and nutrient concentrations and crude‐oil input) are poorly understood. Typically, the analysis of microbial communities includes an initial assessment of the structure of the population using conventional 16S rRNA gene clone libraries and metagenome sequencing 21. Further, the reconstruction of metabolic capacities of microbial communities is performed, but this analysis (using e.g. metatranscriptomics data) is challenging 22. This, in turn, promotes the application of metaproteomics, by which at least the identity and/or the relative number and abundance of expressed proteins can be detected in the environment 23. Simultaneously, a number of approaches have been specifically designed to integrate gene and protein expression data 24, 25 with metabolic fluxes 26, 27, to characterise presumptive active metabolic pathways under different conditions.

Sediment samples were selected for the study for following reasons. The release of thousands of tons of petroleum hydrocarbons (PHs) from anthropogenic activities affects the marine environment and causes severe ecological and economical damage. Once released at the sea surface, PHs are undergoing both weathering processes (evaporation of volatile fraction, photo‐oxidation) and emulsification 12, 13, 14, 15. As a result, significant amount of PHs become heavier, form tar and settle on the sediments 14. Marine sediments are often fine‐grained and the abundance of clay minerals coupled with high organic loads encourages sorption of the most hydrophobic hydrocarbons. Sedimentary accretion can result in burial of hydrocarbons in zones of low redox potential. As a consequence, hydrocarbons have been found in fine‐grained coastal sediments many decades after a spill due to slow anaerobic biodegradation.

In the present paper, we analysed major metabolic patterns in the three distinct oil‐polluted sites of the Mediterranean Sea using an integrated metaproteomics and metabolomics approach. Moreover, based on the proteins being identified, we suggested active metabolic pathways at the organismal level, which is relevant to disentangle the role of each member in the community. Syntrophy between different microbial members in carbon and sulphur cycling has been suggested. A low contribution of microbial members to pollutant catabolism was also established.

## 2 Materials and methods

### 2.1 Study sites

The sampling sites were located along the Northern and Southern Italian coast 16. The investigated sites, in order of latitude, included the following. The first site was the Gulf of Genoa in the northernmost part of the Ligurian Sea in the proximity of the Arenzano Harbour (Genoa, Italy; 44°22'25.75″N, 8°41'59.58″E) where the Tanker *Haven* (*HAV*) sunk in 1991 28. M/T *Haven*, formerly *Amoco Milford Haven*, was a large crude carrier leased to Troodos Shipping. On April 11, 1991, the tanker carrying a cargo load of 144.000 tons of crude oil exploded during a routine operation and spilled about 50.000 tons of petroleum into the Mediterranean. Italian authorities attempted to tow the MT *Haven* closer to shore to reduce the coastal area affected by the oil spill and to improve access to the wreck, but the ship broke into two pieces and sank after burning for three days. After 17 years (in 2008), the Italian Government commissioned the recovery of this area, but the ship and the remaining tar continue to pollute the Mediterranean coasts of Italy and France 28. The second site was the harbour of Messina (*MES*) (Sicily, Italy 38°11'42.27"N, 15°34'25.01"E), a marine harbour that generally suffers chronic petroleum pollution because of intensive maritime traffic and its limited hydrodynamic regimen and restricted area 29, 30. The third site was the harbour of Priolo (*PRI*) Gargallo (Siracusa, Italy; 37°10'27.46"N, 15°12'7.51"E), which is characterised by heavy industrialisation and intensive tanker traffic transporting both crude and refined oil 31. The samples were named based on the code ‘MPS’, which refers to the MetaProteome Source, followed by a short name indicating the origin of the sample as follows: MPS‐HAV (*Haven* Tanker at the Gulf of Genoa); MPS‐MES (the harbour of Messina); and MPS‐PRI (the harbour of Priolo Gargallo).

### 2.2 Environmental measurements and sample collection

Sediment samples (5.0 kg) were collected at a water depth of 1.0 to 78.0 m (June 2011) by scuba. The temperature, salinity, pH, redox potentials and dissolved oxygen were measured by a portable multiparametric probe analyser (WP 600 Series Meters Eutech Instruments Pte Ltd, Singapore). The oxygen concentration was determined using the Winkler method with an automatic endpoint detection burette 716 DNS Titrino (Metrohm AG, Herisau, Switzerland). Samples for measurements of NO_3_^−^, NO_2_^−^ and PO_4_^3−^ were stored at −20°C, and nutrient concentrations were determined later in triplicate in the laboratory using a “SEAL AutoAnalyser QuAAtro” following classical methods 32 with slight modifications adapted for sediments. A conductivity calibration was performed with a KCl 0.01 mol/L control solution. Reference solutions with pH values of 7.0 and 9.0 were employed for the pH metre. The concentrations of microelements were determined through Inductively Coupled Plasma‐Mass Spectrometry (ICP/MS). Ammonium was determined using the indophenol blue technique (IOC, 1983). The dissolved organic carbon content was determined by the dichromate wet oxidation method; the total organic matter content was calculated by multiplying the values of the organic carbon by 1.8. The amount of total extracted and resolved hydrocarbons (TERHC) was determined as follows. Briefly, TERCH were extracted from sediments following the 3550C EPA (Environmental Protection Agency) procedure. In total, a 500 mL mixture of CH_2_Cl_2_:CH_3_COCH_3_ (1:1 v/v) was added to 1000 g of dry sediments and sonicated for 2 min in an ultrasound bath (Branson 1200 Ultrasonic Cleaner, Branson, USA). Samples were further shaken at 150 rpm for 30 min, centrifuged for 10 min at 5000 *g* and the supernatant was passed through a ceramic column filled with anhydrous Na_2_SO_4_ (Sigma‐Aldrich, Milan, Italy). The identical treatment of sediments was repeated with 500 mL of CH_2_Cl_2_, and the obtained solvents were combined and volatilised until dryness. The residues were re‐suspended in CH_2_Cl_2_ prior to the gas chromatography (GC) analysis. All measurements were performed using a Master GC DANI Instruments (Development Analytical Instruments) equipped with a SSL injector and flame ionisation detector (FID). The sample (1 μL) was injected in the splitless mode at 330°C. The analytical column was a Restek Rxi‐5 Sil MS with Integra‐Guard, 30 m x 0.25 mm (ID x 0.25 μm film thickness). The helium carrier gas was maintained at a constant flow of 1.5 mL/min. TERCH were calculated using the mean response factors of *n*‐alkanes, i.e., individual *n*‐alkane concentrations from *n*‐C_15_ to *n*‐C_40_; additionally, pristane and phytane concentrations were calculated for each sample. The amount of TERCH was expressed as ppm (part per million) or mg/kg.

### 2.3 Protein extraction

Sediment samples were subjected to protein isolation using a two‐step protocol. First, before protein extraction, the sediment samples underwent differential centrifugation to eliminate the majority of the crude oil attached to the sediment material (oil interferes with the protein extraction). Therefore, 10.0 g of the sediment samples were mixed with 30.0 mL of sterilised saline solution (5 mM sodium pyrophosphate and 150 mM NaCl) containing Tween 80 (a final concentration of 15 mg/L; Fluka‐Aldrich‐Sigma Chemical Co. (Saint Louis, MO, USA)) in an ice water bath. After re‐suspension, the samples were kept in a water bath ultra‐sonicator (Bandelin SONOREX, Berlin, Germany), maintained at 4ºC on ice, and sonicated (60 W output) for 5 min at 4ºC. The samples were then centrifuged at 100 *g* at 4ºC for 2 min to remove the sediment material. The resulting supernatant was then centrifuged at 13,000 *g* for 15 min at 4ºC to pellet the sediment samples containing microbial cells. The whole‐cell protein extraction was then performed as previously described 33 by heating one volume of mixture of disruption buffer with one volume of the sediment material (obtained as above) at 80ºC for 1 h; during this treatment, the samples were sonicated for 2 min in an ultrasound bath every 10 min. The disruption buffer contained 150 mM NaCl, 2% w/v sodium dodecyl sulphate (SDS), 100 mM ethylenediaminetetraacetic acid (EDTA), 1 M Tris HCl, 100 mM 1,4‐dithio‐D‐threitol (DTT) and a quarter tablet of Complete Protease Inhibitors (Roche Applied Science, Germany) for each 1 mL of buffer. The above procedure was followed by 7 cycles of 5 s sonication in a probe sonicator (Sonicator® 3000; Misonix, Berlin, Germany) and 5 min of centrifugation at 15 000 *g*; the supernatant was spin filtered at 15ºC for 7 h using Vivaspin filters (Sartorius, Germany) with a molecular weight (MW) cut off of 10,000 Da (after 100‐fold SDS dilution using 20 mM triethylammonium bicarbonate buffer (TEAB)). Urea was added prior to spin filtering (a final concentration of 4 M) for a better recovery of proteins 33. Finally, the quantitation of the extracted protein was performed using the Bradford Protein Assay (Bio‐Rad) 34. An average total amount of 150 ± 12 μg total proteins were obtained per each 10 grams of sediment samples, as calculated by Bradford Protein Assay 34.

The heating step at 80ºC was critical for the efficiency of the protein recovery in the sediment samples compared with standard freeze/thaw cycles; this step was also beneficial when extracting proteins from biofilm material 33. No adverse side‐effects (i.e. protein modifications) due to this heating step have previously been reported 33. To avoid oxidation, buffers were degassed before heating steps, and the headspaces of buffers and solutions were flushed with argon. Some deamidation could be observed during quality control, which might be influenced by the extraction protocol and the heating. This was accepted as a tradeoff to ensure lysis/extraction and to prevent enzymatic degradation, but should be monitored in future quantitative studies.

### 2.4 Mass spectrometry and data analysis

Protein solutions were dried using a Concentrator 5301 (Eppendorf, Hamburg, Germany). Further, the protein pellets were solubilised in Laemmli‐buffer prior to 1D SDS‐PAGE for removal of interfering substances and pre‐fractionation. The lanes of a short gel (Supporting Information Figure 1) of approximately 5 cm were cut into ten slices, reduced, carbamidomethylated and subjected to in‐gel tryptic digestion as previously described 35. The purification and de‐salting was performed by ZipTipC18 columns (Merck Millipore, Billerica, MA, USA), resulting in ten fractions per sample. Peptides were reconstituted in 0.1% v/v formic acid, and mass spectrometric measurement was performed using a nanoAcquity ultra performance liquid chromatography (UPLC) (Waters, Milford, MA, USA) coupled linear trap quadrupole (LTQ)‐Orbitrap Velos (Thermo Fisher Scientific, Waltham, MA, USA). Samples were injected with the autosampler and concentrated on a trapping column (nanoAcquity UPLC column, C18, 180 μm × 2 cm, 5 μm, Waters) with water containing 0.1% v/v formic acid at flow rates of 15 μL/min. After 6 min, the peptides were eluted into a separation column (nanoAcquity UPLC column, C18, 75 μm × 15 cm, 1.75 μm, Waters). Chromatography was performed with 0.1% formic acid in solvent A (100% water) and B (100% acetonitrile). The gradient was 2 to 15% B (0‐10 min), 15 to 40% B (10–77 min), 85% B (77–87 min), followed by re‐equilibration at 2% B for 13 min. For an unbiased analysis, a continuous scan of the eluted peptide ions was performed between 300 and 2000 *m*/*z* (automatically switching to MS/MS CID mode on ions exceeding an intensity of 2000) with ten MS/MS events per survey scan. For MS/MS CID measurements, a dynamic precursor exclusion of 120 s was enabled.

To increase the rates of protein identification and to decrease the false negatives rate, the MetaGenomic Sequences (BioProject IDs PRJNA222659 (for MGS‐HAV), PRJNA222657 (for MGS‐MES) and PRJNA222658 (for MGS‐PRI) at NCBI; AZIB00000000 (for MGS‐HAV), AZIC00000000 (for MGS‐MES) and AZIF00000000 (for MGS‐PRI) at DDBJ/EMBL/GenBank) containing a total number of 54,323 unique/non‐redundant sequence entries were complemented by a two‐step database approach 36 as described elsewhere 37. In brief, mass spectra were first searched against prokaryotic entries of the National Center for Biotechnology Information non‐redundant database (NCBInr) using Thermos Proteome Discoverer and Mascot. The unfiltered results (4404 entries) were exported to serve as a complementing database in the main search 37. The final database, a combination of the metagenome and NCBInr sequences, was used for protein identification in MaxQuant (v. 1.5.1.0) 38. Although the use of metagenomic sequences is not necessarily beneficial as previously discussed 23, it was also not possible to know beforehand if a metagenome might be needed or not. For that reason, we combined public databases and metagenomic sequences obtained for the samples herein investigated. Roughly 20% of the proteins were exclusively matched with the metagenomic data derived from these three Mediterranean sites.

The oxidation of methionine was defined as a variable modification, and the carbamidomethylation of cysteine was defined as a fixed modification. The three different sample sets were divided into different parameter groups and matching was disabled. All remaining standard settings were maintained. These included a peptide and protein false discovery rate (FDR) below 1%, at least one peptide, a precursor mass tolerance of 4.5 ppm after mass recalibration and a fragment ion mass tolerance of 0.5 Da. The threshold of only one identified peptide per protein identification was used because FDR controlled experiments counter intuitively suffer from the two‐peptide rule 39. Protein grouping was automatically performed by MaxQuant based on the law of parsimony. The mass spectrometry proteomics data have been deposited to the ProteomeXchange Consortium (http://www.proteomexchange.org) via the PRIDE partner repository 40 with the dataset identifier PXD001490.

Predicted protein sequences were aligned against NCBInr using a BLASTP search. Taxonomic binning of the sequences was performed by summarising the top significant BLASTP hits with e‐values ≤ 0.00001. Additionally, composition‐based binning of contigs containing the genes encoding the identified proteins was performed using the GOHTAM Web‐server 41 to ensure taxonomic affiliations.

### 2.5 Metabolomic fingerprint analysis of sediment samples

The metabolites were extracted from sediment samples as follows. In 100 mL Erlenmeyer flasks, 5 g of sediments were mixed with 10 mL of cold (–80ºC) high‐performance liquid chromatography (HPLC)‐grade methanol (MeOH). The samples were sonicated in a probe sonicator (Sonicator® 3000; Misonix, Berlin, Germany) for 120 sec (at 15 W) in an ice water bath. This procedure was repeated 4 times, and the samples were kept on ice for at least 2 min between each step. After sonication, the supernatant was removed by centrifugation at 10 000 *g* for 30 min at 4ºC, and the supernatant was stored in 20 mL serum vials at –80ºC. The MeOH extracts were filtered using 0.2 μm nylon syringe filters (Thermo Scientific, Rockwood, USA) and analysed by liquid chromatography quadrupole time‐of‐flight mass spectrometry (LC‐Q‐TOF‐MS). The analytical run began with the analysis of Quality Controls (QCs) followed by sampling in a random order; a QC sample was injected in between blocks of four samples until the end of the run. All vials were maintained at 4°C throughout the run. Each metabolic fingerprint was achieved using a liquid chromatography system consisting of a degasser, binary pump, and autosampler (1290 infinity, Agilent; Santa Clara, CA, USA). A total of 0.5 μL was applied to a reverse‐phase column (Zorbax Extend C_18_ 50 × 2.1 mm, 3 μm; Agilent), which was maintained at 60°C during the analysis. The system operated in the positive and negative ion mode at a flow rate of 0.6 mL/min of solvent A (water with 0.1% formic acid) and solvent B (acetonitrile with 0.1% formic acid). The gradient was 5% B (0–1 min), 5 to 80% B (1–7 min), 80 to 100% B (7–11.5 min) and 100 to 5% B (11.5–12 min) followed by re‐equilibration at 5% B for 3 min (15 min of total analysis time). Data were collected in the positive and negative electrospray ionisation (ESI) mode in separate runs on a Q‐TOF (Agilent 6550 iFunnel). Both ion modes operated in the full scan mode (*m*/*z* 50 to 1000 in positive and *m*/*z* 50 to 1100 in negative ion mode). For each mode, the capillary voltage was 3000 V, the scan rate was 1.0 spectra/s, the gas temperature was 250°C, the drying gas flow was 12 L/min, and the nebuliser was 52 psi. The MS‐TOF parameters for the positive ion mode were as follows: fragmentor 175 V, skimmer 65 V and octopole radio frequency voltage (OCT RF Vpp) 750 V. The identical MS‐TOF parameters were used in the negative ion mode, except the fragmentor was set to 250 V. Two reference masses were used for each mode: *m*/*z* 121.0509 ([C_5_H_4_N_4_+H]^+^) and *m*/*z* 922.0098 ([C_18_H_18_O_6_N_3_P_3_F_24_+H]^+^) during the positive analysis and *m*/*z* 112.9855 ([C_2_O_2_F_3_‐H]^−^) and *m*/*z* 1033.9881 ([C_18_H_18_O_6_N_3_P_3_F_24_+TFA‐H]^−^) during the negative analysis. The reference masses were continuously infused into the system to permit constant mass correction. The chromatograms deconvolution and peak integration were performed using Mass Hunter Qualitative Analysis (B.05.00, Agilent).

## 3 Results and discussion

### 3.1 Study sites: physical–chemical characteristics

The rationale behind the sampling strategy was to target sites with aged and chronic contaminations and in diverse environmental locations in the Mediterranean basin. So, the samples herein investigated were inevitably rather heterogeneous (e.g., distinct, conductivity, and NH_4_^+^, PO_4_^3−^, dissolved organic carbon, microelements and O_2_ concentrations and different hydrocarbon loads). However, they are representative for some of the most prevalent types of chronically polluted sites that are distributed within the Mediterranean 16. Moreover, they constitute the basis of a proof‐of‐concept for an integrated approach (metaproteome and metabolome‐based study) to unravel core environmental parameters regulating active microbial populations and activities. As shown in Table 1, at the sampling time the three studied sites exhibited the following most distinct characteristics: (i) a temperature ranging from 15°C (for HAV) to 23ºC (for MES); (ii) a pH from 6.85 (for PRI) to 8.05 (for HAV); (iii) an oxygen concentration ranging from anoxic (for PRI) to 6.0–6.50 mg/L (for HAV); (iv) a conductivity ranging from 49.0 (for HAV and PRI) to 70.0 (for MES) mS/cm; (v) a total concentration of alkane (C_10_–C_40_) of 500 (for MES) to 260 000 (for HAV) mg/kg sediment; (vi) a total concentration of polyaromatic hydrocarbons (PAH) of <1 (for PRI) to 182 (for HAV) mg/kg sediment; (vii) an ammonium concentration ranging from 0.6 (for HAV) to 2.3 (for PRI) μmol/L; (viii) a PO_4_^3‐^ concentration ranging from 0.1 (for HAV) to 0.45 (for PRI) μmol/L; and (ix) a NO_3_^−^concentration ranging from 6 (for HAV) to 29 (for PRI) μmol/L. Values for other parameters are given in Table 1.

**Table 1:** Overall physical–chemical characteristics of the investigated sediment samples

Parameters^a^	HAV	MES	PRI
GPS coordinates	44°22'25.75”N	38°11'42.267"N	37°10'27.462"N
	8°41'59.58"E	15°34'25.014"E	15°12'7.505"E
Depth (m)	78.0	1.0	6.0
C_10_‐C_40_ (ppm)^b^	260 000	500	3922
PAH (ppm)^b^	182	100	<1
Temperature (ºC)	15.0	23.0	19.0
Dissolved O_2_ (mg/L)^c^	6.0–6.5	1.0‐2.2	0
pH	8.05	7.37	6.85
Conductivity (mS/cm)	49.0	70.0	49.0
Ammonium (mkmol/L)	0.6–0.7	7	420
Calcium (mg/L)	420	420	420
PO_4_^3‐^ (mkmol/L)	0.1	0.3	0.45
NO_3_^−^(mkmol/L)	6	8	29
NO_2_^−^ (mkmol/L)	3	2	4
Diss_org_carb (mg/L)^d^	5.00	50.00	125.00
Part_org _carb (μM)^d^	1.40	1.44	1.89
[Microelements] (nM)^e^	392.0	408.0	883.0
Open reading frames (ORF)^f^	8388	40 077	5858
Non‐redundant proteins^g^	310	333	388

a Triplicate measurements were performed with standard deviations lower than 5%.

b Total extracted and resolved petroleum hydrocarbons (TERHC) were extracted and alkanes and polyaromatic hydrocarbons (PAH) determined.

c PRI is an anoxic site; MES is a micro‐aerophilic environment.

d Abbreviations are as follows: Diss_org_carb, dissolved organic carbon; Part_org _carb, particulate organic carbon.

e Microelements include Sc, Cr, Mn, Fe, Ni, Co, As, Se, Mo, Ag, Sn, Sb, Ba, La, Ce, Sm, Eu, Tb, Hf, Au, Hg and heavy metals such as Zn, Cd, Pb and Cu.

f Gene prediction results from sequencing data obtained by Illumina HiSeq and Roche 454 sequencing of metagenomic DNA from the microbial communities in the three polluted sediments collected in the Mediterranean Sea. For accession numbers, see the Materials and Methods section.

g Number of non‐redundant proteins unambiguously identified in the metaproteomes.

The total concentration of hydrocarbons in the sediments (Table 1) exceeded that in clean seawater (15 ppm) by more than 70‐fold. This excess likely occurs because the investigated sites are subjected to chronic petroleum pollution 16.

### 3.2 Metaproteomic analysis: general comments

We selected a shotgun metaproteomic approach to query the active populations within the chronically contaminated sites. The identification depends heavily on protein abundance, and although we are aware that a substantial fraction of the present proteome stays hidden, the identified proteins are assumed to represent the dominant pathways in each system. In total, 651 non‐redundant proteins were unambiguously identified (with a total numbers of proteins of 310 in MPS‐HAV, 333 in MPS‐MES and 388 in MPS‐PRI) (Supporting Information Table 1). Because the proteome analysis could be correlated with the corresponding reference metagenome datasets, the metaproteome sizes were found within a common range observed for other communities and, as it is often the case, a few times smaller than those observed for cultured organisms 42, 43, 44, 45. Although distinct environmental sites were investigated, 106 of 651 proteins (or 16.2%) comprised the subset that was common in all three samples. A total of 90, 143 and 144 sampling site‐specific proteins were identified for HAV, MES and PRI, respectively. This indicated that the HAV, MES and PRI communities displayed overlaps but also considerable heterogeneity at the proteome level and regarding functional categories of identified proteins. The metaproteomic approach applied here, based on the relative number of identified proteins, allowed us to compare the taxonomic annotations and to evaluate the differences between the contributions of particular groups of organisms in the overall communities and to predict the importance of particular sets of proteins for the overall functioning of the community.

### 3.3 Identities of expressed proteins

**Figure 1:**
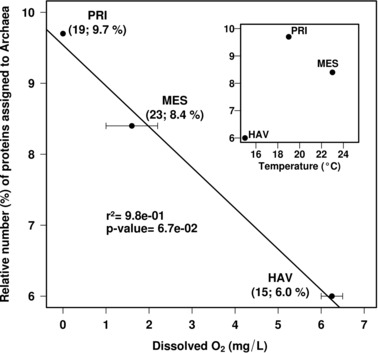
Oxygen concentration as an environmental factor driving the occurrence of proteins assigned to Archaea at the three studied sites. A significant negative correlation (*r* ^2^ = 0.98; *P* = 6.7 × 10^−2^; *t*‐test) was noted between the relative percentage of proteins assigned to Archaea referred to the total number of proteins (to avoid artefacts because of different sample sizes) and the site oxygen concentration. No such correlation was found with other environmental parameters such as site temperature (inset) or other parameters whose values are given in Table 1. The absolute numbers and relative percentage of proteins assigned to Archaea are given in brackets.

**Figure 2:**
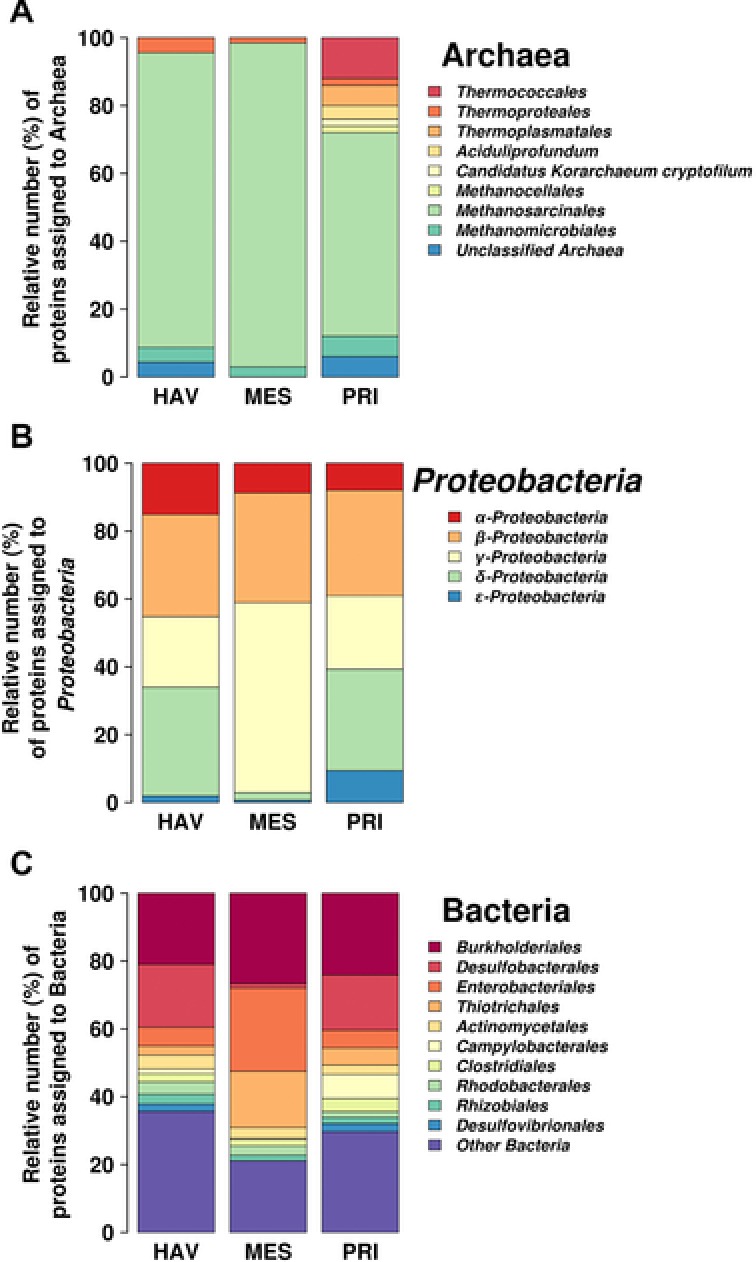
Relative number and distribution of archaeal (A) and bacterial (B, C) proteins in the metaproteomes of marine sediment samples, based on taxonomic bins for proteome‐derived proteins with taxonomic annotation. Distributions in (A) and (C) are at the level of the order, whereas (B) indicates the classes within the phylum *Proteobacteria*.

Taxonomic classifications revealed that the proteins assigned to Bacteria were predominant to a similar extent in all three samples (HAV: 94% (or 291 proteins); MES: 91.6% (or 305); PRI: 90.3% (or 350) of the total proteins). The percentage of proteins binned to Archaea negatively correlated with O_2_ concentration in situ; the highest percentage was obtained at the site with the lowest O_2_ concentration (*r* ^2^ = 0.98; *p*‐value = 0.067) (Fig. 1). By contrast, the relative percentage of archaeal proteins did not correlate with in situ temperature (Fig. 1, inset) as well as crude oil input (or petroleum hydrocarbon concentration [ppm]) or other environmental parameter whose values are given in Table 1. Evidently, the decrease in O_2_ concentrations may stimulate the growth of strictly anaerobic archaea, such as methanogens, thus resulting in the enhanced expression of their proteins. Among all identified archaeal proteins, those associated with the methanogenic *Methanosarcinales* were most numerous in all three sites (HAV: 4.8% (or 14); MES: 6.2% (or 21); PRI: 5.4% (or 21), of the total proteins), as revealed by their higher relative number compared with proteins from other archaeal members (Fig. 2A). In addition to proteins assigned to *Methanosarcinales*, the diversity of archaeal proteins likely originated from *Archaeoglobales*, *Aciduliprofundum, Halobacteriales*, *Thermococcales* and *Thermoplasmatales*. All these organisms were observed in PRI but were missing in HAV and MES (Fig. 2A).

Within the bacterial sub‐proteomes, proteins assigned to the phylum *Proteobacteria* were predominant in all samples (HAV: 66% (or 205); MES: 57% (or 189); PRI: 81% (or 314), referring to total protein numbers). The distribution at the level of classes of *Proteobacteria* (Fig. 2B) revealed only significant differences in the number of proteins assigned to *Deltaproteobacteria*, displaying significantly lower protein expression levels in MES when compared to HAV (8.4‐fold, in terms of numbers identified) and PRI (9.5‐fold). The proteins assigned to *Epsilonproteobacteria* were more numerous in PRI than in HAV (7.2‐fold) and MES (11.6‐fold) (Fig. 2B). Because PRI is an anaerobic environment (Table 1), the higher numbers of proteins from *Epsilonproteobacteria* most likely reflect their association with anoxic sites; notably, members of this class have also been found to be abundant in pollutant‐degrading microbial consortia operating under aerobic conditions 43, 46. However, the proteins assigned to *Gammaproteobacteria* were detected at higher number in MES than in HAV (2.8‐fold) and PRI (2.6‐fold) (Fig. 2B). In addition to proteins from the phylum *Proteobacteria*, the proteins likely derived from *Firmicutes* (HAV: 7.8% (or 24); MES: 6.2% (or 21); PRI: 8.1% (or 27), of the total proteins) and *Acidobacteria* (HAV: 5.8% (or 18); MES: 4.4% (or 15); PRI: 4.8% (or 19), of the total proteins) formed the second and third predominant groups. Additional groups of Bacteria that expressed proteins at identifiable quantities in our assay are summarised in Fig. 2C. These findings were verified by a phylogenetic analysis of NCBInr derived peptides using the web‐based Unipept tool 47. Note, that no identified proteins in our dataset were affiliated with the genus of typical specialised hydrocarbonoclastic (HCB) bacteria 5, 9, 10, other than *Cycloclasticus* in HAV. This may correlate with the fact that they were not found in the sediment samples (except those assigned to *Cycloclasticus* in HAV), as revealed by SSU rRNA hypervariable tag analysis; see original non‐chimeric SSU rRNA hypervariable tag 454 sequences that are archived at the EBI European Read Archive under accession number PRJEB5322, for details.

Among all proteins, the ten functional groups associated to the most numerous identified proteins were the following (Supporting Information Table 1): (i) ABC transporters or outer membrane proteins (116, or 18% of the total), (ii) hypothetical proteins (109, or 17%); (iii) ribosomal proteins (32, or 5%); (iv) AprAB adenosine‐5‐phosphosulfate reductases (22, or 3.4%); (v) DsrAB sulfite reductases (18, or 2.8%); (vi) TonB‐dependent receptors (14, or 2.2%); (vii) chaperones (14 or 2.2%); (viii) glutamate decarboxylases/dehydrogenases/synthases (13, or 2%); (ix) ATP synthases (9, or 1.4%); and (x) proteins for the C_1_‐compounds uptake and methanogenesis [9 methyl‐coenzyme M reductases, 7 methanol dehydrogenases and 7 methanol‐5‐hydroxybenzimidazolylcobamide methyltransferases; or 3.5%]). These functional groups suggest that apart from the transport and energy production systems, the metabolism of sulphur, C_1_‐compounds and glutamate are among the most active functions within all communities, albeit at different levels. These metabolisms have been commonly found to be markedly stimulated under anaerobic conditions 48, 49. Proteins involved in glutamate conversion were only found in HAV and PRI (11 and eight non‐redundant proteins, respectively), but not in MES; the fact that glutamate metabolism is generally activated in response to low temperature stress 50 and that both HAV and PRI were characterised by a much lower sea‐water temperature compared to MES (Table 1), suggests that low temperature in combination with anaerobic stress might be responsible for the noted differences. As mentioned, a high number of proteins (109, or 17% of the total) were identified with a hypothetical role, suggesting that their function could be associated with anaerobic conditions. However, insights into their physiological roles remain limited, and further experimental evidence is needed.

### 3.4 Differences at the metabolic and organismal levels as revealed through metaproteomics

Recent studies have utilised the proteomics approach to determine active metabolic pathways operating in microbial communities 23. Here, on the basis of their probable functions, we were able to capture overall functional differences of the three chronically polluted sites at the level of metabolic pathways (specifically, in relation to sulphur and C_1_ metabolism) and hypothesised different pathway organisations at the organism level. These differences are detailed below.

**Figure 3:**
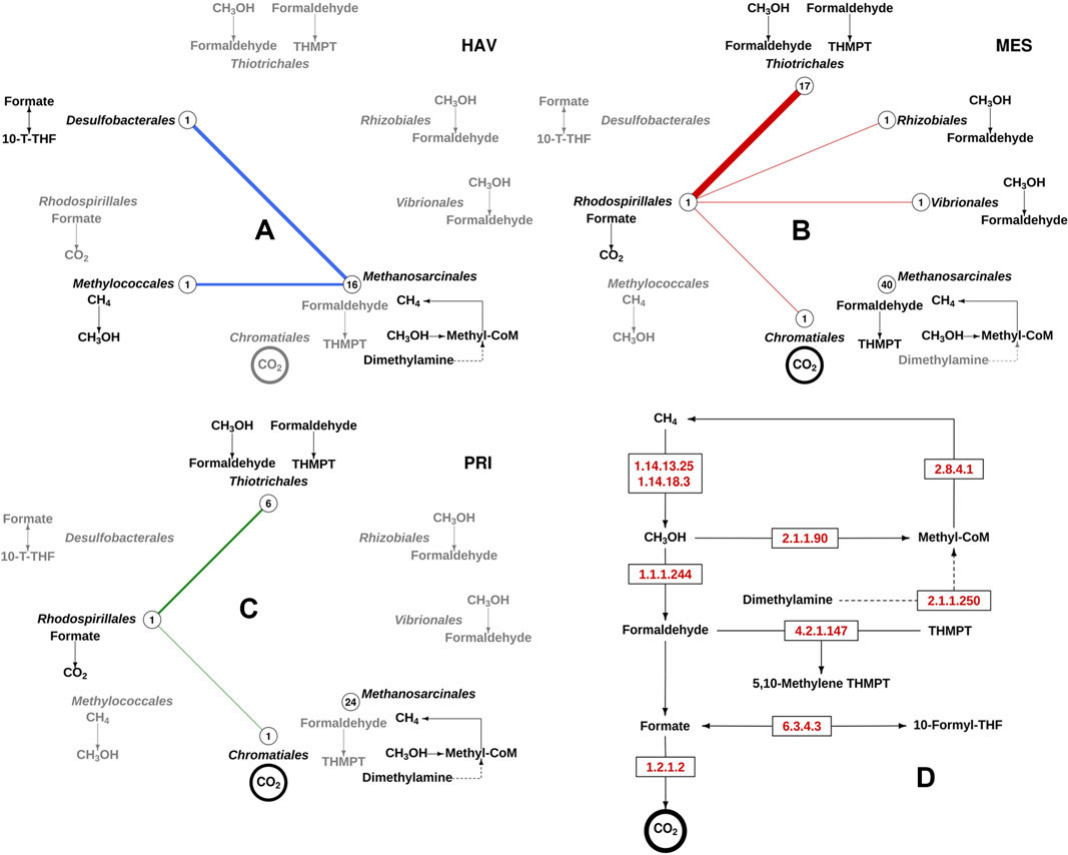
Reconstruction of the C_1_ metabolism at the organismal level in the microbial communities inhabiting petroleum‐polluted marine sediments based on the proteome analysis. Panels A, B and C, represent the active pathways in HAV, MES and PRI, respectively. The complete C_1_ metabolism that included the metabolic coupling of CH_4_ and CH_3_OH metabolism and the Wood‐Ljungdahl pathway is shown in Panel (D). As shown, only a small portion of the reactions within the entire C_1_ metabolism (D) was identified as being active in HAV, MES and PRI (A–C). The presence of enzymes for each transformation and the taxonomic affiliation of polypeptides are shown. Circles in A–C represent the relative number of proteins (referred to the total number proteins assigned to these pathways) in a sample assigned to each taxonomic group (the total number of proteins of each is indicated in the circles). Solid lines (HAV, blue; MES, red; PRI, green) in A–C display the syntrophy between different members as they participate in contiguous reactions, as described in panel (D). The relative number of proteins assigned to these syntrophic reactions is presented by the line thickness. In panels (A–C), the grey colour indicates transformations for which no proteins in the proteome were identified, whereas the black colour represents transformations for which proteins were found (putatively active reactions). Transformations in the CH_4_ and CH_3_OH metabolism for which no proteome evidence were found (e.g., the glutathione (GSH) pathway connecting methanol with CO_2_) are not indicated. Note: the representation of CO_2_ in a circle (bottom of each panel) represents the presence of a carbon dioxide concentrating carboxysome shell protein, a structural protein involved in CO_2_ accumulation within the cells, but with no catalytic function.

*C_1_ metabolism*: A total of 31 proteins (or 5.1% of the total proteome) potentially involved in the CH_4_, CH_3_OH and CO metabolism (HAV: 11; MES: 20; PRI: 19) were detected (Supporting Information Table 1; Fig. 3). This corresponds to 23% of the theoretical number of proteins (*in silico* proteome) presumptively involved in C_1_ metabolism, as determined after examining the potential protein‐coding genes (≥ 20 amino acids long) from the meta‐sequences (see accession number in Section 2.4). Protein signatures for the initial step of CH_4_ conversion to CH_3_OH were only found in HAV; this conversion can be performed by methane monooxygenase from bacterial members of the order *Methylococcales* (the top hit BAH22843, beta‐subunit, PmoB). A possible explanation for the presence of methane monooxygenase for methanotrophy in HAV but not in PRI and MES may be due to the presence of a higher accumulation of petroleum hydrocarbons in HAV (Table 1) and thus the presence of difficult‐to‐degrade alkanes and aromatics 12.

Enzymatic pathways for C_1_‐compounds uptake and (methylotrophic) methanogenesis were detected in all three communities, although to different extents (Supporting Information Table 1; Fig. 3). The evidence for this pathway were the presence of: (i) methanol corrinoid proteins, methanol‐5‐hydroxybenzimidazolylcobamide methyltransferases andmethylcobalamin:coenzyme M methyltransferases converting CH_3_OH to methyl‐CoM; and (ii) methyl‐coenzyme M reductases. In addition, trimethylamine:corrinoid methyltransferases that convert trimethylamine to methyl‐CoM and dimethylamine, were also found in the proteomes. Notably, all these enzymes were unambiguously attributed to *Methanosarcinales*.

**Figure 4:**
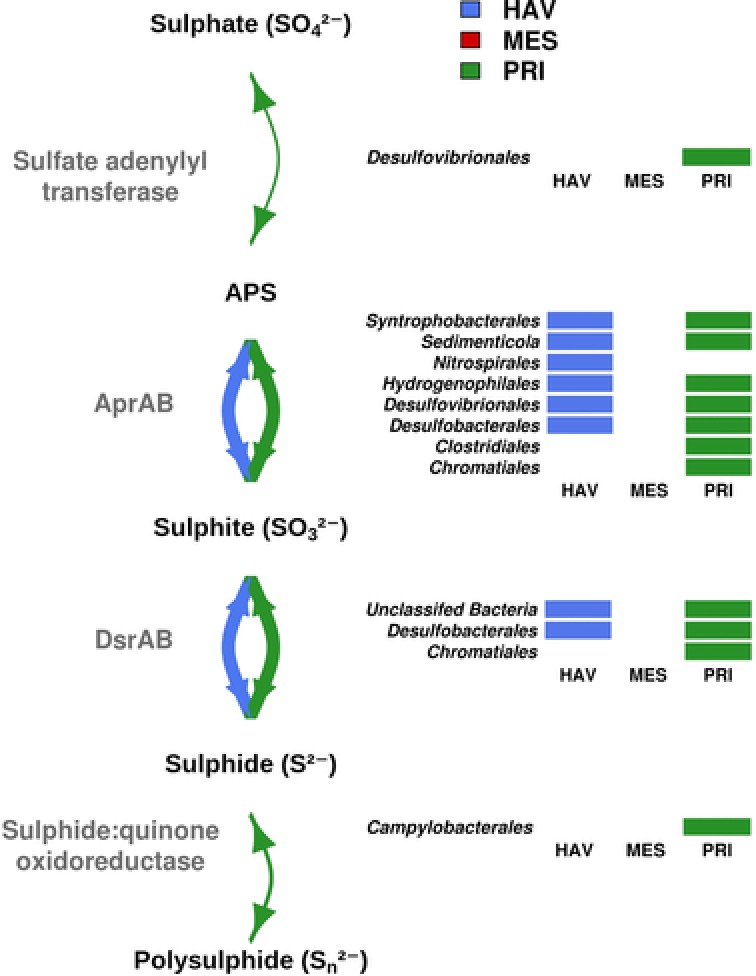
Reconstruction of the sulphur metabolism patterns in microbial communities inhabiting petroleum‐polluted marine sediments based on the proteome analysis. The presence of enzymes for each transformation (linked by solid lines) and the taxonomic affiliation of polypeptides are shown. The thickness of the solid lines represents the relative number of enzymes (referred to the total number proteins assigned to these pathways) associated to each of the transformations in the pathway. Transformations in the sulphur metabolism for which no proteome evidence were found (e.g., sulphur assimilation metabolism via ATP sulfurilase) are not indicated.

Signatures for methanol‐utilising bacterial methylotrophs were also identified in the proteomes. Proteins for the subsequent conversion of CH_3_OH to formaldehyde by methanol dehydrogenase were only detected in MES and PRI (Fig. 3). *Proteobacteria*, notably those associated to members of the orders *Thiotrichales* (in MES and PRI),*Vibrionales* (in MES) and *Rhizobiales* (in MES), were presumably capable of the utilisation of CH_3_OH (Fig. 4). Methanol conversion to formaldehyde was not evident in HAV proteome data. Enzymes for the subsequent metabolism of formaldehyde were found in MES and PRI, namely formaldehyde‐activating enzymes that transform formaldehyde to tetrahydromethanopterin (THMPT). Notably, the occurrence of the latter conversion was supported by proteins assigned to *Methanosarcinales* (in MES and PRI) and *Thiotrichales* (in PRI). No signatures for the conversion of formaldehyde to formate were observed, although a formate dehydrogenase (binned to *Rhodospirillales*) converting formate to CO_2_ was found in MES and PRI. A carbon monoxide dehydrogenase (CODH catalytic subunit; top hit WP_031450384.1) (binned to *Desulfobacterales*) for the carbonyl branch of the Wood‐Ljungdahl pathway was found in PRI (Fig. 3). Finally, a carbon dioxide concentrating mechanism/carboxysome shell protein (top hit WP_015107688.1), a structural protein (with no catalytic function and no Enzyme Nomenclature [EC] number) involved in the storage of enzymes participating in CO_2_ metabolism, such as carbonic anhydrase and RubisCO 51, was found in both MES and PRI.

Taken together, the presence of two active processes is suggested: methylotrophic methanogenesis mediated by Archaea (*Methanosarcinales*) and methanol/formaldehyde detoxification by a set of bacteria. In addition, all studied communities possess highly developed trophic networks based on assimilatory and dissimilatory metabolism of C_1_‐compounds.

The taxonomy‐guided pathway reconstruction emphasised differences at the organism level in relation to the CH_4_ and CH_3_OH metabolism. Thus, Fig. 3 shows the contribution of major organisms in all three sites, in which a clear pathway partitioning and metabolic coupling between community members are discernible. The metabolism and activation of CH_4_ seems to be supported by a bacterial member of the *Methylococcales* order. Finally, the further methylotrophic conversion of CH_3_OH was dominated by bacterial proteins (from members of the orders *Thiotrichales*, *Vibrionales*, *Rhizobiales*, *Rhodospirillales* and *Desulfobacterales*) and, to some extent, by archaeal proteins from methylotrophic methanogens of *Methanosarcinales*.

*Sulphur metabolism, energy conservation and detoxification*: A total of 54 proteins (or 8.3% of the total) that were potentially involved in the metabolism of sulphur compounds were detected (Supporting Information Table 1; Fig. 4). While these proteins were numerous in HAV (28) and PRI (45), they were absent in MES (Fig. 4). This is particularly noticeable given that the metagenome sequence data set of sample MES was 4.8 and 6.9‐fold bigger compared with those of samples HAV and PRI, respectively (Table 1). The absence of these proteins as revealed in the MPS‐MES proteome reflects their low biological significance in MES. In fact, 27 potential protein‐coding genes (≥ 20 amino acids long) presumptively involved in sulphur metabolism were found in the metagenomic sequences of MES (see the accession number in Section 2.4), all of which had the protein expression levels below our detection limit (Supporting Information Table 1; Fig. 4).

We detected AprAB (adenosine‐5‐phosphosulfate reductases) and DsrAB (sulfite reductases converting sulphite (SO_3_^2−^) to adenylyl sulphate (or facilitating the reverse reaction) and sulphide (H_2_S)) both in HAV (23 proteins in total) and PRI (36 proteins in total) (Fig. 4). Such genes have also been detected in the Deepwater Horizon deep‐sea plume at numbers higher than in non‐plume samples, as sulphite reduction has been likely coupled with hydrocarbon degradation 12. A total of 15 AprAB proteins conformed to the common set in the two sites. The proteins in HAV were assigned to *Desulfobacterales* (15), *Desulfovibrionales* (2), *Nitrospirales* (1), *Sedimenticola* (1) and *Syntrophobacterales* (2). Proteins in PRI were assigned to *Desulfobacterales* (21), *Chromatiales* (5), *Syntrophobacterales* (3), *Clostridiales* (2), *Desulfovibrionales* (2), *Hydrogenophilales* (2) and *Sedimenticola* (1). The presence of protein signatures for active sulphite reduction in HAV and PRI, agrees also with the identification of ten heterodisulfide reductases in both sediments (five each) but not in MES; they were assigned to *Desulfobacterales* (Supporting Information Table 1). Such proteins could provide electrons for sulphite reduction, although, in analogy with methanogenic archaea, it was also speculated that they can also support the generation of a proton motive force 52.

One sulfate adenylyltransferase converting sulphate (SO_4_^2−^) to adenylyl sulphate (binned to *Desulfovibrionales*), two sulfide:quinone oxidoreductases converting *n* sulphide (*n* HS^−^) to polysulphide, a defensive HS‐oxidation pathway implying the dump of excess electrons from the cytoplasm/membrane (binned to *Campylobacterales*), and a rhodanese involved in the detoxification of cyanide (CN^−^) (binned to *Methanosarcinales*), were only found in PRI. Taken together, the PRI microbial community contained high number of proteins involved in various metabolic pathways of both oxidised and reduced sulphur intermediates. Note that, in contrast to sulphite reduction (see above), gene transcripts for the oxidation and detoxification of sulphur compounds were neither found in the Deepwater Horizon deep‐sea plume 12, nor in HAV and MES, suggesting the environmental settings in PRI could particularly enrich for those processes.

### 3.5 Community metabolic activity associated with biodegradation

Only one relevant protein, a carboxymuconolactone decarboxylase (EC: 4.1.1.4) involved in the protocatechuate catabolism, was identified in the metaproteomes (Supporting Information Table 1). The protein, identical to EGD01850 from *Burkholderiales* (100% sequence identity), was only found in HAV and PRI. This suggests that the examined metaproteomes contained minimal signatures for active biodegradation pathways, possibly because of the low rates of biodegradation processes in chronically petroleum‐polluted sediments that are limited by low oxygen (or the presence of other forms of organic matter). This environment encouraged the prevalence of proteins involved in the metabolism of C_1_–compounds, suggesting the latter segment of the carbon cycle is more active under the given conditions.

Metabolome profiling might be more sensitive in the detection of presumptive chemical signatures indicating biodegradation capabilities of petroleum constituents. Our protocol comprised of the isolation of metabolites obtained from sediment material followed by a metabolome‐wide scan via a combination of mass spectrometry (MS) with liquid chromatography (LC) separation. A total of 1485 (LC‐MS negative mode) and 3390 (LC‐MS positive mode) mass features were found in each analysis after deconvolution (Supporting Information Table 2). Empirical formulas were assigned to accurate masses with a maximum error of 5 ppm using a CEU Mass Mediator (http://biolab.uspceu.com/mediator), and putative chemical species were identified.

Only 24 out of 4776 metabolite mass features (or 0.5% of the total) were tentatively attributed as pollutants or chemical intermediates, thus suggesting that the accumulation of those compounds occurred at a low level, reflecting low degradation rates. It is therefore important to evaluate whether such subtle contribution can be considered to fall within a common range. It is notable, however, that no report to date has described the metabolomic profiling of microbial communities in other contaminated marine sediments; therefore, little is known about whether the observed results are within a common range. The fact that HCB are the specialists for the degradation of alkanes and polycyclic aromatic hydrocarbons (PAH) 5, and that those were absent in our datasets (see Fig. 2), together with the lower rates of degradation under anoxic conditions, may agree with the lack of detection of such chemical signatures in the three investigated sites at a higher level. In addition to that, it is noteworthy that protein signatures for phosphorous uptake and metal reduction, often stimulated by oil contamination 12, were not found in our datasets. This suggests that petroleum oil biodegradation in the chosen experimental sites occurs at a lower level than in the recently reported studies on Deepwater Horizon or other contaminated sites.

Having said that, we are fully aware about the limitations of MS which may introduce a bias certain due to the secondary reactions in the ion source and collision reactions in the MS and that the identification was tentative. Additionally, to ensure the metabolite identity, pure reference compounds for MS/MS analysis are needed in further experiments to unambiguously identify the substrates/pollutants and the degradation intermediates.

## 4 Concluding remarks

In conclusion, by examining the metaproteomes and metabolomes of three chronically polluted sites, we found that the examined sites contained only minimal signatures of active biodegradation pathways, possibly because of the low rates of biodegradation processes in chronically petroleum‐polluted sediments that are limited by the presence of low oxygen (or presence of other forms of bioavailable organic matter). This environment encouraged the prevalence of proteins involved in the metabolism of C_1_–compounds, suggesting the latter segment of the carbon cycle is more active under the given conditions. Our study highlights the presence of methanotrophic bacteria that can oxidise methane through sequential reactions catalysed by a series of enzymes including methane monooxygenase, methanol dehydrogenase, formaldehyde dehydrogenase and formate dehydrogenase. This full conversion was suggested to be active only in the HAV sampling site, which corresponds to the highest concentration of oxygen. MES and PRI did not show evidence for active methane oxidizers, but rather displayed a high diversity of methanol‐consuming bacteria, which was also observed in HAV. Methanol, which may be produced as an intermediate of organic matter (and hydrocarbon) biodegradation, can thus be used by prokaryotic communities inhabiting all three sites as another source of carbon and energy 53. Our results have revealed the presence of microorganisms presumably active in methane oxidation to be uniquely present in HAV. The first step in the aerobic methane oxidation was only found in HAV, agreeing with the higher O_2_ concentration in this site compared with MES and PRI. Additionally, all three sediments contained microorganisms that utilise C_1_ compounds such as methanol. Accordingly, the ability to utilise methanol and select other single carbon compounds (but not methane) was found in MES and PRI, whereas PRI and HAV were populated by trimethylamine‐utilising microbes. Our results (Fig. 3 showing the simplified metaproteomics‐based reconstruction of methane and methanol metabolism) displayed the contributions of different archaeal and bacterial groups and their link to active methane and methanol cycling (three groups in HAV and six in MES and four in PRI). Among the eight groups involved in those metabolisms, only one group (*Methanosarcinales*) was found in all three samples. Figures 3 and 4 depict possible microbial metabolic networks and also point at syntrophic interactions. Seemingly, individual members of the network could operate only a small part of the particular pathway. We hypothesise that the identified proteins do represent enzymes for predominant reactions and that corresponding transformations may be complementary to those supported by other community members making, therefore, the entire pathway complete. Furthermore, the results of this study draw attention to the yet untapped diversity of proteins from archaea and bacteria operating in the Mediterranean Sea sediments under conditions of limited oxygen concentrations.

*The authors have declared no conflict of interest*.

## Supporting information

As a service to our authors and readers, this journal provides supporting information supplied by the authors. Such materials are peer reviewed and may be re‐organized for online delivery, but are not copy‐edited or typeset. Technical support issues arising from supporting information (other than missing files) should be addressed to the authors.

Supporting Information Figure 1 SDS‐PAGE: Coomassie‐stained SDS‐PAGE of the protein extracts from HAV, MES and PRI sediments.

Supporting Information Table 1 Proteins of HAV, PRI and MES communities identified by the metaproteomic approaches. Protein annotations are shown. Panel (A) shows the overall proteome raw data. Panel (B) includes the list of the identified proteins with the corresponding best hits, protein annotation, presumptive taxonomic affiliation and number of peptides per protein. Panel (C) included the list of proteins specifically found in relation to CH4, CH3OH, CO and sulphur metabolism.

Supporting Information Table 2 Metabolite mass featuresputatively identified and semi‐quantified by metabolomic approaches by LC‐MS (‐) and LC‐MS (+) in the sediment samples. For differential quantitative metabolomics, we compared the metabolomes of sediment samples by evaluating the peak area from the chromatographic peaks. A list of the masses identified by LC‐MS using positive and negative polarities following alignment are presented for samples HAV, MES and PRI. Because the samples interact during the separation technique and MS, quality controls (QCs) must be employed during LC‐MS to ensure analytical reproducibility. QC samples are required throughout the analytical runs at periodic intervals to monitor variations in signal across time and at the beginning of the sequence to stabilise the system [1]. QC samples were prepared for LC‐MS by pooling and mixing equal volumes of each sample. After gently vortexing, the mix was also filtered and subsequently transferred to an analytical vial and analysed. In all cases, the technique (LC‐MS positive (+) or negative (–) ionisation mode), mass (in Da) and retention time (RT) (in minutes) (as Da@min), and the abundance level per sample are shown. Panel abbreviations and content are as follows: LC(‐) total and LC(+) total are lists of significantly different masses obtained after alignment in the LC‐MS using the negative (‐) and positive (+) polarities, respectively.
