# Supplementary material for: Metaproteomics and metabolomics analyses of chronically petroleum‐polluted sites reveal the importance of general anaerobic processes uncoupled with degradation
Source: Proteomics. 2015 Aug 27;15(20):3508–20. doi: 10.1002/pmic.201400614 (PMC4973819; doi:10.1002/pmic.201400614)
Supplement: Supplementary file 1 — Supporting Information Figure 1 SDS‐PAGE: Coomassie‐stained SDS‐PAGE of the protein extracts from HAV, MES and PRI sediments. [file PMIC-15-3508-s001.docx]

**Supporting Material**

**Supporting Information Figure 1** SDS-PAGE: Coomassie-stained SDS-PAGE of the protein extracts from HAV, MES and PRI sediments.

**
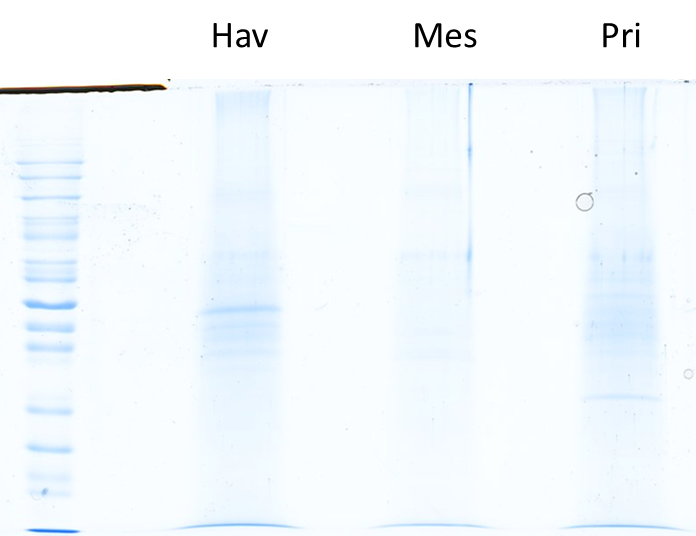
**

**Supporting Information Table 1** Proteins of HAV, PRI and MES communities identified by the metaproteomic approaches. Protein annotations are shown. Panel (A) shows the overall proteome raw data. Panel (B) includes the list of the identified proteins with the corresponding best hits, protein annotation, presumptive taxonomic affiliation and number of peptides per protein. Panel (C) included the list of proteins specifically found in relation to CH_4_, CH_3_OH, CO and sulphur metabolism.

**Supporting Information Table 2** Metabolite mass features putatively identified and semi-quantified by metabolomic approaches by LC-MS (-) and LC-MS (+) in the sediment samples. For differential quantitative metabolomics, we compared the metabolomes of sediment samples by evaluating the peak area from the chromatographic peaks. A list of the masses identified by LC-MS using positive and negative polarities following alignment are presented for samples HAV, MES, and PRI. Because the samples interact during the separation technique and MS, quality controls (QCs) must be employed during LC-MS to ensure analytical reproducibility. QC samples are required throughout the analytical runs at periodic intervals to monitor variations in signal across time and at the beginning of the sequence to stabilise the system [1]. QC samples were prepared for LC-MS by pooling and mixing equal volumes of each sample. After gently vortexing, the mix was also filtered and subsequently transferred to an analytical vial and analysed. In all cases, the technique (LC-MS positive (+) or negative (-) ionization mode), mass (in Da) and retention time (RT) (in minutes) (as Da@min), and the abundance level per sample are shown. Panel abbreviations and content are as follows: LC(-) total and LC(+) total are lists of significantly different masses obtained after alignment in the LC-MS using the negative (-) and positive (+) polarities, respectively.

**Supporting References**

1. [Dunn, W. B](http://www.ncbi.nlm.nih.gov/pubmed?term=Dunn%20WB%5BAuthor%5D&cauthor=true&cauthor_uid=21720319)., [Broadhurst, D](http://www.ncbi.nlm.nih.gov/pubmed?term=Broadhurst%20D%5BAuthor%5D&cauthor=true&cauthor_uid=21720319)., [Begley, P](http://www.ncbi.nlm.nih.gov/pubmed?term=Begley%20P%5BAuthor%5D&cauthor=true&cauthor_uid=21720319)., [Zelena, E](http://www.ncbi.nlm.nih.gov/pubmed?term=Zelena%20E%5BAuthor%5D&cauthor=true&cauthor_uid=21720319). *et al*., Procedures for large-scale metabolic profiling of serum and plasma using gas chromatography and liquid chromatography coupled to mass spectrometry. Nat. Protoc. 2011, 6, 1060-1083.
